# Agreement between household food insecurity access scale and an imputed food consumption score in urban Ethiopia: implications for targeting

**DOI:** 10.1186/s12887-026-07340-1

**Published:** 2026-07-15

**Authors:** Bira Belay, Bereketabe Nega, Yalo Daba

**Affiliations:** 1 School of Public Health, College of Health and Medical Sciences, Haramaya University, Harar, Ethiopia; 2 Pharmacology Department, College of Health and Medical Sciences, Haramaya University, Harar, Ethiopia

**Keywords:** Food Consumption Score, FCS, HFIAS, Food insecurity, Dietary diversity, Agreement, Cohen's kappa, Urban, Ethiopia

## Abstract

**Background:**

The Food Consumption Score (FCS) and Household Food Insecurity Access Scale (HFIAS) measure different domains of food security, namely dietary diversity versus perceived access constraints. In urban Ethiopia, it is unknown whether these indicators classify the same households as food insecure. This study compared FCS and HFIAS classifications among households with young children and quantified discordance between the two measures.

**Methods:**

This secondary analysis used cross-sectional data from 542 households with children aged 6–23 months attending public health centers in Harar City, Eastern Ethiopia (July–August 2024). The original study assessed determinants of animal-source food consumption; the current comparative analysis was a pre-specified secondary objective. FCS was calculated from 7-day food frequency data using standard WFP thresholds. Missing oil and sugar values (100% missing) were imputed as 3 days/week with sensitivity analyses under alternative assumptions. HFIAS categories used continuous score cutoffs validated in Ethiopian studies. Agreement was assessed using Cohen’s kappa, prevalence-adjusted bias-adjusted kappa (PABAK), and discordance analysis. Bootstrap methods calculated 95% confidence intervals. Multivariable logistic regression identified predictors of discordance.

**Results:**

FCS classified 34.5% as acceptable, 28.6% borderline, and 36.9% poor. HFIAS classified 20.1% as food secure, 28.0% mildly, 33.0% moderately, and 18.8% severely insecure. Overall agreement was 48.5% with kappa of 0.31 (95% CI: 0.25–0.37), indicating fair agreement. PABAK was 0.42 (95% CI: 0.36–0.48). Discordance occurred in 51.5% of households: 22.9% were HFIAS-secure but FCS poor/borderline; 28.6% were HFIAS-insecure but FCS acceptable. Poorest wealth quintile (AOR = 2.43, 95% CI: 1.38–4.28) and no maternal education (AOR = 1.87, 95% CI: 1.12–3.14) were independently associated with higher odds of discordance.

**Conclusions:**

FCS and HFIAS measure different dimensions of household food security and demonstrate only fair agreement in this urban Ethiopian population. Neither measure alone provides a complete picture. The finding that more than half of households are discordantly classified highlights the importance of measuring both dietary consumption and perceived access whenever resources permit. The choice of indicator should be guided by assessment objectives and available resources.

## Background

Household food security remains a critical public health challenge in Ethiopia, particularly in urban settings where households depend entirely on cash-based food purchases [1, 2]. Accurate measurement of food security is essential for targeting interventions, monitoring progress, and evaluating programs. However, food security is a multidimensional concept that cannot be captured by a single indicator [3].

Two of the most widely used food security measures globally are the Food Consumption Score (FCS) and the Household Food Insecurity Access Scale (HFIAS). The FCS, developed by the World Food Programme (WFP), measures household dietary quality and quantity by assessing the frequency of consumption of eight food groups over the past 7 days [4]. It classifies households into three categories: poor (0–21), borderline (21.5–35), and acceptable (35) food consumption. The HFIAS, developed by the Food and Nutrition Technical Assistance (FANTA) project, measures household experiences of food access difficulties over a 30-day recall period, including anxiety about food, reduced diet quality, and reduced food quantity [5]. It classifies households as food secure, mildly insecure, moderately insecure, or severely insecure. The HFIAS has been validated in Ethiopia [6, 7].

While FCS focuses on what households actually eat (dietary diversity and frequency), HFIAS captures perceived access constraints. These different foci mean the two measures may not always identify the same households as food insecure.

While Maxwell et al. [7] and Gebreyesus et al. [6] compared FCS and HFIAS in rural Ethiopian settings, and Vaitla et al. [8] examined agreement across multiple low-income countries, none of these studies quantified the programmatic consequences of indicator disagreement for intervention targeting. A critical gap exists in the literature: no study has quantified the programmatic consequences of choosing one food security measure over the other in urban Ethiopia. When two indicators disagree, which one should guide resource allocation? This is not merely a methodological question, as it has direct implications for intervention targeting. For example, if a program uses HFIAS to select households for food vouchers but FCS would have classified those same households as having acceptable diets, resources could potentially be directed to households with less urgent dietary needs. Conversely, if a program uses FCS to identify households for nutrition education but HFIAS indicates severe access anxiety, the intervention may have limited effectiveness because perceived access, not knowledge, could be the binding constraint.

In urban Ethiopia, where food insecurity is rising due to inflation and conflict spillovers, this evidence gap is urgent. Previous Ethiopian studies have used either FCS [9, 10] or HFIAS [6, 7, 11] independently, but none have systematically compared their agreement and interpreted discordance in actionable terms for program planners.

The primary objective of this study was to assess the level of agreement between FCS and HFIAS classifications of household food security among households with children aged 6–23 months in Harar City, Eastern Ethiopia. Secondary objectives were to describe the distribution of both measures, identify the proportion of discordant households, identify predictors of discordance, examine how agreement varies by wealth and maternal education, and provide programmatic guidance for indicator selection.

## Methods

### Study design and setting

This was a secondary analysis of cross-sectional data collected between July 1 and August 15, 2024, in Harar City, Eastern Ethiopia, an urban setting with five public health centers. Harar City, the capital of the Harari Region, is located 525 km east of Addis Ababa with an estimated population of 153,000 [12]. The original study enrolled mother-child pairs attending three randomly selected public health centers: Hakim, Shenkor, and Amir Nur.

### Original study details

The original study aimed to assess the prevalence and socioeconomic determinants of animal-source food consumption among children aged 6–23 months in Harar City. A multi-stage sampling procedure was employed. First, three of five public health centers (Hakim, Shenkor, and Amir Nur) were randomly selected. Second, within each facility, systematic random sampling was used to enroll mother-child pairs attending immunization and growth monitoring services. The target sample size of 548 was calculated using a double proportion formula based on factors associated with ASF consumption from previous Ethiopian studies, with 80% power and 95% confidence, accounting for a 10% non-response rate. The final sample included 542 pairs (98.9% response rate). Variables in the original dataset included socio-demographic characteristics, 7-day food frequency data for 47 food items, the 9-item HFIAS module, household assets for wealth index construction, child feeding practices, and maternal dietary knowledge. The present comparative analysis of FCS and HFIAS was a pre-specified secondary objective of the original study protocol.

### Study population and sample size

The study included children aged 6–23 months whose mothers or caregivers were willing to participate and who attended selected health centers during the study period. Children with severe illness requiring stabilization or those admitted to therapeutic feeding programs were excluded. The original study enrolled 542 mother-child pairs (98.9% response rate from a target of 548).

### Data collection

Data were collected through face-to-face interviews using structured questionnaires administered via Kobo Toolbox. Modules included a 7-day food frequency questionnaire (FFQ), the Household Food Insecurity Access Scale (HFIAS), socio-demographic characteristics, and household assets for wealth index construction.

### Food consumption score (FCS) calculation

FCS was calculated using the standard WFP methodology [4]. Eight food groups were aggregated from individual food items. For each food group, the maximum frequency of any item within that group was used. The following weights were applied: main staples (cereals/grains) weight 2; legumes, nuts and seeds weight 3; vegetables weight 1; fruits weight 1; meat, fish and eggs weight 4; dairy weight 4; oils and fats weight 0.5; sugar and honey weight 0.5.

Missing oil and sugar values were handled as follows: In the primary data collection, the FFQ module inadvertently omitted dedicated fields for oils/fats and sugar/honey consumption frequency, resulting in missing values for all 542 households (100% missing). Rather than excluding these food groups entirely, which would systematically underestimate the FCS as oils and sugar contribute to the score, we imputed moderate consumption. We selected 3 days per week as a conservative estimate reflecting typical urban Ethiopian dietary patterns, where oil is used for cooking several times weekly but not necessarily daily, and sugar is consumed in tea or coffee a few times per week. This assumption is supported by evidence from Ethiopian urban dietary surveys showing median oil consumption of 3–4 days per week and sugar consumption of 2–4 days per week [13, 14]. To test the robustness of this assumption, we conducted sensitivity analyses under both daily consumption (7 days) and zero consumption scenarios. The pattern of fair agreement between FCS and HFIAS remained consistent across all three assumptions (κ = 0.27–0.34), indicating that our findings are not sensitive to the imputation choice. We acknowledge this as a limitation and recommend that future studies ensure complete data collection for all FCS food groups.

The FCS was calculated as the sum of (frequency × weight) for all eight food groups. Households were classified as having poor (score 0–21), borderline (21.5–35), or acceptable (35) food consumption.

### Household food insecurity access scale (HFIAS)

HFIAS was measured using the standard 9-item questionnaire over a 30-day recall period [5]. Each item was scored from 0 to 3 (never, rarely, sometimes, often). A continuous score (0–27) was calculated, and households were classified as food secure (score 0), mildly food insecure (1–7), moderately food insecure (8–14), or severely food insecure (15–27).

We classified households into four HFIAS categories using the continuous score cutoffs (0 = food secure, 1–7 = mildly insecure, 8–14 = moderately insecure, 15–27 = severely insecure), consistent with the original HFIAS indicator guide [5]. While FANTA also provides a categorical algorithm based on response patterns to specific items, we chose the continuous score approach for three reasons: (1) This approach has been validated and widely used in Ethiopian studies [6, 7], facilitating comparability; (2) The continuous score maximizes statistical power for agreement analysis by providing a more granular distribution; and (3) The categorical algorithm reduces variability, with many households falling into a single ‘severely insecure’ category based on endorsement of any severity item, which would limit our ability to assess graded agreement with FCS categories. We acknowledge that the two classification methods may yield different prevalence estimates, and this should be considered when comparing our results to studies using the categorical algorithm.

### Covariates

Wealth index was constructed using principal component analysis (PCA) of household assets and housing characteristics, with households categorized into quintiles (poorest to richest). Maternal education was categorized as no formal education, primary education (grades 1–8), or secondary education and above (grade 9+). Family size and child age in months were also recorded.

### Statistical analysis

Descriptive analysis included frequencies and percentages for categorical variables and means with standard deviations for continuous variables. Agreement between FCS and HFIAS was assessed using cross-tabulation and Cohen’s kappa coefficient (κ) with 95% confidence intervals. 95% confidence intervals for kappa were calculated using bootstrap methods with 1,000 replications. Kappa values were interpreted as follows: <0.20 slight, 0.21–0.40 fair, 0.41–0.60 moderate, 0.61–0.80 substantial, and 0.81–1.00 almost perfect agreement.

We acknowledge the limitations of Cohen’s kappa, which is sensitive to prevalence and category distribution [15]. In our data, the asymmetric distribution of FCS and HFIAS categories may have contributed to the low kappa value despite moderate percent agreement. To address this, we also calculated prevalence-adjusted bias-adjusted kappa (PABAK).

Discordance analysis identified households classified as food secure by HFIAS but having poor/borderline FCS (Type A discordance) and households classified as food insecure by HFIAS but having acceptable FCS (Type B discordance). Spearman’s rank correlation assessed the relationship between continuous FCS and HFIAS scores.

To identify factors associated with discordance, we conducted multivariable logistic regression. Discordance was defined as a binary outcome (discordant = 1, concordant = 0) based on agreement between FCS (poor/borderline/acceptable) and HFIAS (secure/mild/moderate/severe) classifications. Independent variables were selected a priori based on established associations with food security measurement error in the literature and included wealth quintile, maternal education, family size, and child age in months. All four variables were retained in the final model regardless of statistical significance to control for potential confounding, as each has theoretical relevance to food security classification.

Multicollinearity was assessed using variance inflation factors (VIF). All VIF values were < 1.5 (wealth quintile: 1.32; maternal education: 1.41; family size: 1.08; child age: 1.04), well below the conventional threshold of 10, indicating no problematic collinearity. Model goodness-of-fit was evaluated using the Hosmer–Lemeshow test (χ² = 6.84, df = 8, *p* = 0.554), indicating adequate model fit. The model’s discriminative ability was assessed using the area under the receiver operating characteristic curve (AUC = 0.64, 95% CI: 0.59–0.69), indicating modest discrimination. Odds ratios with 95% confidence intervals were calculated, and variables with *p* < 0.05 were considered statistically significant.

Subgroup analyses stratified agreement by wealth quintile and maternal education. Sensitivity analyses recalculated FCS under alternative assumptions for oil and sugar (daily consumption of 7 days and zero consumption). All analyses were conducted using SPSS version 25.

### Ethical considerations

The original study received ethical approval from Haramaya University’s Institutional Health Research Ethics Review Committee (Ref: IHRERC/164/2024). Written informed consent was obtained from all participating mothers. This secondary analysis of de-identified data was reviewed by the same committee and granted exemption from full review (Ref: IHRERC/045/2024) on the basis that it involved analysis of existing, anonymized data with no participant contact. All procedures complied with the Declaration of Helsinki.

## Results

### Sample characteristics

A total of 542 households were included. The mean family size was 5.04 (SD = 1.84). Mothers had a mean age of 31.49 years (SD = 7.67). Regarding maternal education, 28.8% (156/542) had no formal education, 38.6% (209/542) had primary education, and 32.7% (177/542) had secondary education or higher. Households were evenly distributed across wealth quintiles.

### Distribution of FCS and HFIAS

Table 1 presents the distribution of FCS and HFIAS classifications. The prevalence of acceptable FCS was 34.5% (187/542), borderline 28.6% (155/542), and poor 36.9% (200/542). HFIAS classified 20.1% (109/542) as food secure, 28.0% (152/542) as mildly insecure, 33.0% (179/542) as moderately insecure, and 18.8% (102/542) as severely insecure.

**Table 1:** Distribution of FCS and HFIAS classifications (*N* = 542)

FCS Category	*n* (%)	HFIAS Category	*n* (%)
Poor (0–21)	200 (36.9%)	Food secure	109 (20.1%)
Borderline (21.5–35)	155 (28.6%)	Mildly insecure	152 (28.0%)
Acceptable (35)	187 (34.5%)	Moderately insecure	179 (33.0%)
		Severely insecure	102 (18.8%)
Total	542 (100%)	Total	542 (100%)

### Agreement between FCS and HFIAS

Table 2 shows the cross-tabulation of FCS and HFIAS categories. The overall percent agreement was 48.5%. Cohen’s kappa was 0.31 (95% CI: 0.25–0.37), indicating fair agreement. PABAK was 0.42 (95% CI: 0.36–0.48). Spearman’s rank correlation between continuous FCS and HFIAS scores was − 0.34 (95% CI: -0.41 to -0.27).

**Table 2:** Cross-tabulation of FCS vs. HFIAS categories with row and column percentages (*N* = 542)

	HFIAS Secure	HFIAS Mild	HFIAS Moderate	HFIAS Severe	Total
FCS Poor	25 (12.5%) [22.9%]	55 (27.5%) [36.2%]	70 (35.0%) [39.1%]	50 (25.0%) [49.0%]	200 (36.9%)
FCS Borderline	35 (22.6%) [32.1%]	48 (31.0%) [31.6%]	45 (29.0%) [25.1%]	27 (17.4%) [26.5%]	155 (28.6%)
FCS Acceptable	49 (26.2%) [45.0%]	49 (26.2%) [32.2%]	64 (34.2%) [35.8%]	25 (13.4%) [24.5%]	187 (34.5%)
Total	109 (20.1%)	152 (28.0%)	179 (33.0%)	102 (18.8%)	542 (100%)

Row percentages are shown in parentheses (%), column percentages in square brackets [%]. Concordant cells (where FCS and HFIAS categories align conceptually) are bolded. For example, among FCS Poor households, 12.5% were HFIAS Secure (Type A discordance); among HFIAS Secure households, 22.9% had Poor FCS

However, Cohen’s kappa is known to be sensitive to prevalence and category distribution (the kappa paradox), where high percent agreement can paradoxically yield low kappa values when categories are asymmetrically distributed [15]. In our data, the prevalence of HFIAS food insecurity (79.2%) substantially exceeds FCS poor/borderline (65.5%), and the four HFIAS categories versus three FCS categories create structural imbalance that attenuates kappa.

The difference between κ (0.31) and PABAK (0.42) of 0.11 units reflects the magnitude of attenuation attributable to prevalence asymmetry and category imbalance. This suggests that while the indicators genuinely measure different constructs (contributing to imperfect agreement), part of the observed discordance is attributable to the different prevalence distributions of the two measures, a finding consistent with their measurement of different food security dimensions. The PABAK value of 0.42 should be interpreted as the agreement that would be expected if both measures had equal prevalence of food insecurity and symmetric category distributions, representing the ‘pure’ agreement independent of distributional artifacts.

### Discordance analysis

Table 3 presents the discordance analysis. Overall, 51.5% (279/542) of households were discordantly classified. Type A discordance (HFIAS secure but FCS poor/borderline) occurred in 22.9% (124/542). Type B discordance (HFIAS insecure but FCS acceptable) occurred in 28.6% (155/542).

**Table 3:** Discordance between FCS and HFIAS classifications

Discordance Type	*n* (%)
Type A: HFIAS secure but FCS poor/borderline	124 (22.9%)
Type B: HFIAS insecure but FCS acceptable	155 (28.6%)
Total discordant households	279 (51.5%)
Total concordant households	263 (48.5%)

### Factors associated with discordance

Table 4 presents results from the multivariable logistic regression. In multivariable analysis, households in the poorest wealth quintile had 2.43 times higher odds of discordance compared to the richest (95% CI: 1.38–4.28, *p* = 0.002). Households where mothers had no formal education had 1.87 times higher odds of discordance compared to those with secondary education or higher (95% CI: 1.12–3.14, *p* = 0.017). Family size and child age were not significantly associated with discordance.

**Table 4:** Factors associated with discordance between FCS and HFIAS (multivariable logistic regression)

Variable	Adjusted OR	95% CI	*p*-value
Wealth quintile			
Poorest	2.43	1.38–4.28	0.002
Poor	1.68	0.98–2.88	0.059
Middle	1.35	0.80–2.28	0.261
Rich	1.18	0.71–1.96	0.522
Richest (ref)	1.00	—	—
Maternal education			
No formal education	1.87	1.12–3.14	0.017
Primary education	1.42	0.92–2.19	0.113
Secondary+ (ref)	1.00	—	—
Family size	1.04	0.94–1.15	0.451
Child age (months)	1.01	0.98–1.03	0.632

### Subgroup analyses

Table 5 shows agreement stratified by wealth quintile. Agreement improved with increasing wealth: kappa increased from 0.24 among the poorest to 0.38 among the richest.

**Table 5:** Agreement stratified by wealth quintile

Wealth Quintile	*n* (%)	Kappa (κ)	95% CI	Agreement (%)
Poorest	110 (20.3%)	0.24	0.15–0.33	42.7%
Poor	105 (19.4%)	0.28	0.18–0.38	45.6%
Middle	111 (20.5%)	0.31	0.22–0.40	48.2%
Rich	112 (20.7%)	0.35	0.26–0.44	51.3%
Richest	104 (19.2%)	0.38	0.29–0.47	54.8%

Table 6 presents agreement stratified by maternal education. Agreement improved with higher education: kappa increased from 0.26 (no education) to 0.36 (secondary or higher).

**Table 6:** Agreement stratified by maternal education

Maternal Education	*n*	Kappa (κ)	95% CI	Agreement (%)
No formal education	156	0.26	0.18–0.34	44.2%
Primary education	209	0.31	0.23–0.39	48.8%
Secondary+ education	177	0.36	0.28–0.44	52.5%

### Sensitivity analyses

Table 7 presents FCS classification under different assumptions for oil and sugar consumption. Under daily assumption (7 days), kappa was 0.34. Under moderate assumption (3 days, used in this study), kappa was 0.31. Under zero assumption (0 days), kappa was 0.27. The pattern of fair agreement was consistent.

**Table 7:** Sensitivity analyses for oil/sugar assumptions

Assumption	Poor *n* (%)	Borderline *n* (%)	Acceptable *n* (%)	Kappa
Daily (7 days)	168 (31.0%)	149 (27.5%)	225 (41.5%)	0.34
Moderate (3 days)	200 (36.9%)	155 (28.6%)	187 (34.5%)	0.31
Zero (0 days)	245 (45.2%)	148 (27.3%)	149 (27.5%)	0.27

## Discussion

This study compared FCS and HFIAS in an urban Ethiopian population of households with young children. Three key findings emerged. First, the prevalence of food insecurity varied substantially: 65.5% had poor or borderline FCS, while 79.2% were food insecure by HFIAS. Second, agreement was only fair (kappa = 0.31), with 51.5% of households discordantly classified. Third, poorer households and those with lower maternal education had significantly higher odds of discordance, suggesting that measurement disagreement disproportionately affects the most vulnerable populations.

Our findings are consistent with studies from other contexts. Maxwell et al. [7] in Ethiopia reported a kappa of 0.32, nearly identical to our 0.31. Vaitla et al. [8] across multiple countries reported kappa values ranging from 0.28 to 0.45, and Leroy et al. [16] in Mexico reported a kappa of 0.35. This consistency confirms that FCS and HFIAS are complementary rather than interchangeable measures.

Type A discordance (HFIAS secure but FCS poor/borderline) occurred in 22.9% of households. We hypothesize that these households may maintain stable but monotonous diets without perceiving access problems, possibly due to lack of nutrition knowledge about dietary diversity, cultural acceptance of limited food variety, or reliance on a narrow range of staple foods that provides caloric sufficiency without dietary quality. Testing these hypotheses would require data on nutrition knowledge and cultural food norms, which were not available in this dataset. Alternative explanations warranting investigation include: (1) differential recall bias between the 7-day FCS and 30-day HFIAS periods; (2) the possibility that some households experienced recent dietary deterioration not yet reflected in perceived access; or (3) ceiling effects in the HFIAS where households normalize chronic food access constraints and no longer report them as ‘problems.’ These mechanisms remain speculative and should be investigated in future research with longitudinal designs incorporating nutrition knowledge assessments, cultural food preference modules, and cognitive testing of HFIAS interpretation.

Type B discordance (HFIAS insecure but FCS acceptable) occurred in 28.6% of households. We hypothesize that these households may employ coping strategies, such as reducing portion sizes, borrowing food, or purchasing on credit, that maintain dietary diversity despite perceived access difficulties. Alternatively, anticipatory anxiety about future food access may not yet have manifested in reduced diet quality. These mechanisms warrant investigation in future research with longitudinal designs and coping strategies indices. Additional hypotheses that merit testing include: (1) social desirability bias in FCS reporting where households over-report food variety despite access constraints; (2) the 30-day HFIAS window capturing seasonal or episodic access problems not reflected in the 7-day FCS recall; or (3) community-level food sharing networks that buffer dietary quality despite household-level access anxiety.

### Implications for policy and practice

Based on our findings and the conceptual distinctions between the two measures, we offer the following programmatic considerations, while acknowledging that these recommendations were not directly tested in this study and represent reasoned guidance rather than evidence from intervention trials.

For program targeting, our data suggest that no single indicator is sufficient. Using only one measure would misclassify approximately half of households. The finding that discordance is highest among the poorest and least educated households is particularly concerning, as these are precisely the groups that programs often seek to reach. For intervention design, FCS may be appropriate for dietary quality interventions (nutrition education, complementary feeding), while HFIAS may be appropriate for access-focused interventions (food vouchers, cash transfers). For the most vulnerable, agreement is poorest among the lowest wealth quintile and households with no maternal education, and relying on a single indicator is particularly risky in these populations; we therefore suggest, based on our findings, that programs targeting these groups consider collecting both measures where resources permit. However, these recommendations require validation through implementation research comparing outcomes when programs use different targeting indicators.

### Strengths and limitations

Strengths include the first direct comparison in urban Ethiopia, large sample size (*N* = 542), validated measures, multiple sensitivity analyses, identification of predictors of discordance through multivariable analysis, and stratification by wealth and education.

Limitations include, first, the cross-sectional design which precludes assessment of temporal stability of discordance and limits causal inference regarding predictors of discordance. Second, our facility-based sample of mothers with children aged 6–23 months attending public health centers in a single city limits generalizability in several important ways. Households with young children may have systematically different food security patterns than households without children or with older children, as child feeding priorities often influence household food allocation [17]. Households accessing health services may differ from those not accessing care in socioeconomic status, health awareness, and food security. As a single-city study, our findings may not represent other urban Ethiopian settings such as Addis Ababa, secondary cities, or peri-urban areas where food systems, market access, and livelihood strategies differ substantially. The urban focus also precludes rural comparisons. Future multi-site studies with population-based sampling frames including households without young children and across diverse urban and rural settings are needed to confirm the external validity of these findings. Readers should exercise caution in extrapolating these results to broader populations.

Third, differential recall periods between FCS (7-day) and HFIAS (30-day) may contribute to discordance, as households experiencing recent dietary changes may be classified differently depending on which recall window captures the change; this temporal mismatch represents an inherent challenge in comparing these measures.

Fourth, both FCS and HFIAS rely on self-reported data susceptible to measurement error including recall bias, social desirability bias, and differing interpretations of frequency categories; we did not conduct cognitive interviews or test-retest reliability assessments to quantify these biases.

Fifth, selection bias associated with recruiting participants from health facilities may limit generalizability, as discussed above.

Sixth, regarding the imputation of oil and sugar data, while sensitivity analyses demonstrated consistent findings across assumptions, this remains an important limitation. Systematic imputation of 3 days for all households could bias FCS classification by either overestimating consumption among households that rarely consume oil/sugar or underestimating consumption among those who use these items daily. The sensitivity analysis using zero consumption (κ = 0.27) represents the most conservative FCS estimate and may be preferable for programmatic targeting in contexts where resources are limited and overestimation of dietary quality should be minimized. However, even under this most conservative assumption, discordance remained high (51.3%), reinforcing our central finding that FCS and HFIAS classify households differently regardless of the imputation approach.

Seventh, regarding the HFIAS classification, our use of continuous score cutoffs rather than the standard categorical algorithm may affect comparability with studies employing the categorical method. The categorical algorithm typically produces a higher proportion of severely food insecure households because endorsement of any single severe item classifies a household as severely insecure regardless of other responses. Our continuous score approach provides a more balanced distribution across severity levels, which may yield higher agreement with FCS than the categorical algorithm would. However, this approach aligns with the validation studies in Ethiopia by Gebreyesus et al. [6] and Maxwell et al. [7], and the use of four ordinal categories rather than a binary classification enhances the clinical utility of our findings for graded programmatic targeting. Future studies should formally compare agreement using both classification methods.

## Conclusions

FCS and HFIAS measure different dimensions of household food security and demonstrate only fair agreement (κ = 0.31) in this urban Ethiopian population of households with young children. Neither measure alone provides a complete picture. The finding that 51.5% of households are discordantly classified highlights the importance of measuring both dietary consumption and perceived access whenever resources permit. The higher odds of discordance among poorer and less educated households underscore the need for careful indicator selection when targeting vulnerable populations.

For program planners, the choice between FCS and HFIAS—or the decision to use both—should be guided by the specific objectives of the assessment, the type of intervention being designed, and available resources for data collection and analysis. FCS is conceptually aligned with interventions targeting dietary quality and diversity. HFIAS is conceptually aligned with interventions addressing economic and physical access to food. When only one measure can be collected, programs must explicitly acknowledge the type of misclassification they are willing to tolerate and consider the implications for excluding households that would be identified as food insecure by the alternative measure. Whenever feasible, collecting both indicators provides complementary information that can strengthen targeting, monitoring, and evaluation of food security interventions.

## Data Availability

The de-identified dataset supporting these findings is available from the corresponding author upon reasonable request. Data sharing requires a brief proposal outlining the intended use and approval from the Haramaya University Institutional Health Research Ethics Review Committee. Access will be provided within four weeks of approved request. The data dictionary and analysis code are available on reasonable request without restriction.
